# Diagnosis, treatment and follow-up of patients with acromegaly in a clinical practice setting in Spain: the ACROPRAXIS program Delphi survey

**DOI:** 10.1007/s11102-019-01012-3

**Published:** 2019-12-10

**Authors:** Pedro de Pablos-Velasco, Eva María Venegas, Cristina Álvarez Escolá, Carmen Fajardo, Paz de Miguel, Natividad González, Ignacio Bernabéu, Nuria Valdés, Miguel Paja, Juan José Díez, Betina Biagetti

**Affiliations:** 1 Endocrinology Service, Hospital Universitario de Gran Canaria Dr. Negrín, C/Barranco de la Ballena, s/n, 35010 Las Palmas de Gran Canaria, Spain; 2 Endocrinology Service, Hospital Universitario Virgen del Rocío, Seville, Spain; 3 Endocrinology Service, Hospital Universitario La Paz, Madrid, Spain; 4 Endocrinology Service, Hospital Universitario de La Ribera, Valencia, Spain; 5 Endocrinology Service, Hospital Universitario Clínico San Carlos, Madrid, Spain; 6 Endocrinology Service, Hospital Universitario Virgen Macarena, Seville, Spain; 7 Endocrinology Service, Hospital Clínico Universitario Santiago de Compostela, Santiago de Compostela, Spain; 8 Endocrinology Service, Hospital Universitario Central de Asturias, Oviedo, Spain; 9 Endocrinology Service, Hospital Universitario de Basurto, Bilbao, Spain; 10 Endocrinology Service, Hospital Universitario Puerta de Hierro Majadahonda, Madrid, Spain; 11 Endocrinology Service, Hospital Universitario Vall d’Hebrón, Barcelona, Spain

**Keywords:** Acromegaly, Clinical practice, Guidelines, Patient management

## Abstract

**Aim:**

The ACROPRAXIS program aims to describe the management of acromegaly in Spain and provide guidance.

**Methods:**

Ninety-three endocrinologists were organized into 13 panels to discuss the practical issues in managing acromegaly. Based on the key learnings, an online Delphi survey with 62 statements was performed, so those statements achieving consensus could be used as guidance. Statements were rated on a 9-point scale (9, full agreement; consensus > 66.6% of response in the same tertile).

**Results:**

Ninety-two endocrinologists (98.8%) answered two rounds of the survey (mean age 47.6 years; 59.8% women; median 18.5 years of experience). Consensus was achieved for 49 (79%) statements. *Diagnosis*: The levels of insulin-like growth factor I (IGFI) is the preferred screening test. If IGFI levels 1–1.3 ULN, the test is repeated and growth hormone (GH) after oral glucose tolerance test (OGTT) is assessed. A pituitary magnetic resonance is performed after biochemical diagnosis. *Treatment*: Surgery is the first treatment choice for patients with microadenoma or macroadenoma with/without optical pathway compression. Pre-surgical somatostatin analogues (SSA) are indicated when surgery is delayed and/or to reduce anaesthesia-associated risks. After unsuccessful surgery, reintervention is performed if the residual tumor is resectable, while if non-resectable, SSA are administered. *Follow*-*up* First biochemical and clinical controls are performed 1–3 months after surgery. Disease remission is considered if random GH levels are < 1 µg/L or OGTT is < 1 or ≤ 0.4 µg/L, depending on the assay’s sensitivity.

**Conclusion:**

Current clinical management for acromegaly is homogeneous across Spain and generally follows clinical guidelines.

## Introduction

Acromegaly is a disease characterized by growth hormone (GH) hypersecretion, usually due to a pituitary adenoma, which causes disproportionate skeletal, soft tissue, and organ growth, resulting in multisystem-associated comorbidities, including hypertension, diabetes, sleep apnea, arthropathy, and increased mortality [1, 2]. There are no pathognomonic features and the symptoms progress insidiously; thus, the diagnosis is usually delayed (a median of 10 years in women and 8 years in men) [3, 4]. The average age at presentation ranges from 40 to 50 years old [4], and the most frequent presenting signs/symptoms are changes in physical appearance (dysmorphic features and excessive growth of hands and feet), headache, fatigue/asthenia, sweating, sleep apnea, and menstrual disturbances in women [3]. Diagnosis is confirmed by high levels of GH and insulin-like growth factor I (IGFI), which provides a measure of integrated GH secretion [5–7]. Treatment includes three different therapies: surgery, medical treatment (somatostatin analogues [SSA], the human GH receptor antagonist pegvisomant, and the dopaminergic agonist cabergoline) and radiotherapy (RT) [5–7].

When and how to apply the different treatment modalities, as well as the specific GH and IGFI values and measurement method for diagnosis, plus the patient management during follow-up are detailed in the available acromegaly guidelines [5–7]. However, in acromegaly, as in other diseases, real life practice does not always follow clinical guidelines [8]. In this regard, the implementation of acromegaly guidelines to the clinical practice in Spain might be limited in certain hospitals due to logistical-related issues, and thus, it was of interest to describe the current Spanish clinical practice. Once described, the common practices could be submitted to a Delphi survey and those achieving consensus could be used to provide local guidance for the management of acromegaly, while taking under consideration the current state of healthcare and clinical practice in Spain (ACROPRAXIS program). Due to the diversity of resources access and clinical practices in Europe, sharing this information might be of interest to compare the practice of other groups in other countries.

## Materials and methods

The ACROPRAXIS program was conducted in two phases. In the first phase, we aimed to define the current clinical practice in Spain. A scientific committee of 11 expert endocrinologists developed an online questionnaire with 45 questions about the diagnosis, treatment and follow-up of acromegaly. The questionnaire was anonymously answered by 93 endocrinologists, with at least 5 years of experience in acromegaly, from different geographic areas of Spain, grouped in 13 region panels. The results were discussed by the panelists in regional meetings, with a special attention to those issues in which the reported clinical practice was less homogeneous or was different from the guidelines´ recommendations.

Based on the conclusions of the regional meetings, the scientific committee gathered the common practices on the management of acromegaly, which, in the second phase of the program, had to be validated by all the panelists following the Delphi methodology (a reliable technique for reaching a consensus among a panel of experts) [9]. The online Delphi survey consisted on 62 statements regarding diagnosis (18 statements), treatment (25) and follow-up (19). The 93 participants were asked to rate each statement anonymously on a Likert-like scale from 1 (“completely disagree”) to 9 (“completely agree”). Responses were grouped by tertiles: 1–3: Disagreement (D), 4–6: Indeterminate (I) (no agreement or disagreement), 7–9: Agreement (A). Consensus (C) on a statement was reached when the responses of 2/3 or more participants (≥ 66.6%) were in the same tertile as the median value of all the reported responses for that statement.

### Statistical analysis

A descriptive analysis of all items using the mean (± standard deviation), median (interquartile range; IQR), and minimum and maximum values was performed. The Kolmogorov–Smirnov test was used to check for goodness of fit of the data to a normal distribution.

The internal consistency of the questionnaire was measured by the Cronbach’s alpha (Cα), which can range between 0 and 1, from lower to greater reliability (acceptable values: > 0.7, high reliability: 0.7–0.9, very high reliability > 0.9) [10]. In addition, inter-rater reliability was assessed by the intra-class correlation coefficient (r_i_) (poor: r_i_ < 0.40, fair: r_i_ = 0.40–0.59, good: r_i_ = 0.60–0.74, and excellent: r_i_ = 0.75–1.0) [11].

The correlation between the two rounds of the questionnaire was measured by the Spearman coefficient (r_s_) (none or poor: r_s_ = 0–0.25, weak: r_s_ = 0.26–0.50, moderate to strong: r_s_ = 0.51–0.75, and strong to very strong: r_s_ = 0.76–1) [12]. The Kappa index (k) was calculated to estimate the qualitative agreement between the rounds having into account the three answer groups (1–3, 4–6 and 7–9) (none or poor: k < 0.20, weak = 0.21 to 0.40, moderate: k = 0.41 to 0.60, good: k = 0.61 to 0.80, and very good: k = 0.81 to 1) [13]. All these values were calculated for the overall survey and for each of the three blocks (diagnosis, treatment and follow-up). Statistical significance was considered when p < 0.05.

The variation coefficient (VC) of the questionnaire was calculated for every round, along with the delta or relative increase in the second round above the first (VC second-VC first/VC first). When delta is < 10%, there is no large variability between the rounds, and thus, there is no need for another round.

## Results

Ninety-two (98.9%) of the 93 panelists who participated in the local meetings responded to the first and second rounds of the survey. Out of these, 59.8% were women, mean age was 47.6 ± 8.6 years, with a median of 18.5 years (IQR: 10–55) of experience in the management of acromegaly.

The survey had high internal consistency (Cα > 0.8) and inter-rater reliability (r_i_ > 0.7), overall and in each of the blocks (Table 1). There was no great variability between the two rounds of the survey (relative increase of VC = 6.5%), and thus, a third round was not necessary.

**Table 1:** Survey consistency

	Round 1		Round 2	
	Cα (p)	r_i_ (p)	Cα (p)	r_i_ (p)
TOTAL (62 items)	0.918 (< 0.001)	0.864 (< 0.001)	0.923 (< 0.001)	0.865 (< 0.001)
Diagnosis block (18 items)	0.807 (< 0.001)	0.789 (< 0.001)	0.807 (< 0.001)	0.789 (< 0.001)
Treatment block (25 items)	0.825 (< 0.001)	0.797 (< 0.001)	0.894 (< 0.001)	0.819 (< 0.001)
Follow-up block (19 items)	0.846 (< 0.001)	0.805 (< 0.001)	0.848 (< 0.001)	0.805 (< 0.001)

Cα: Cronbach’s alpha; r_i_: intra-class correlation coefficient

The Spearman coefficient (r_s_ = 0.985, p < 0.001 for the overall survey) and Kappa index (k = 0.962, p < 0.001 for the overall survey) values were high, due to the low number of statements modified in round 2 with respect to round 1.

In the first round, consensus was not achieved on 15 (24.2%) statements. Only 3 out of those 15 statements were resubmitted to a second round. The rest were not resubmitted since they described clinical practice, which would not have changed in subsequent rounds. Two of the non-consensus resubmitted statements were reformulated (S22 and 26), since it seemed they were not clear, and the third one was resubmitted without modifications (S51), since consensus for this statement was almost reached in the first round and it was of interest to see if panelists wanted to reconsider their position. All three statements passed to a second round without specifying the previous consensus percentage achieved.

After the second round, consensus was obtained for 49 (47 agreement and 2 disagreement) of the 62 statements (79%): 15 (83.3%) in the diagnosis block (Table 2), 19 (76%) in the treatment block (Table 3), and 15 (78.9%) in the follow-up block (Table 4).

**Table 2:** Diagnosis block

	Statement	Median (P25–P75)	Median range	Participants in median range, n (%)	Consensus	Guidelines*
1	The screening test of choice that I use for acromegaly in case of clinical suspicion is the determination of IGFI	9 (9–9)	7–9	92 (100%)	C-A	Y
2	I consider that IGFI is of choice for the diagnosis of acromegaly in patients with diabetes	8 (7–9)	7–9	71 (77.2%)	C-A	Y
3	If IGFI values range between 1 and 1.3 ULN, I repeat the IGFI determination	8 (7–9)	7–9	77 (83.7%)	C-A	X
4	If IGFI values range between 1 and 1.3 ULN, I determine GH after OGTT	8 (7–9)	7–9	74 (80.4%)	C-A	X
5	After biochemical confirmation of acromegaly, I perform a pituitary MRI with gadolinium	9 (9–9)	7–9	92 (100%)	C-A	Y
6	In the presence of a baseline GH value between 0.4 and 1 μg/L and IGFI > 1.3 and < 2 ULN, I determine GH after OGTT	9 (8–9)	7–9	85 (92.4%)	C-A	Y
7	Within the study of comorbidities in acromegaly, I include a general biochemistry test and a complete assessment of pituitary reserve	9 (9–9)	7–9	92 (100%)	C-A	Y
8	Within the study of comorbidities in acromegaly, I perform a routine colonoscopy	9 (7–9)	7–9	72 (78.3%)	C-A	Y
9	Within the study of comorbidities in acromegaly, I include a routine thyroid echography	6 (4–8)	4–6	33 (35.9%)	NC- I	N
10	Within the study of comorbidities in acromegaly, I include an echocardiogram	8 (6–9)	7–9	65 (70.7%)	C-A	Y
11	Within the study of comorbidities in acromegaly, I include routine polysomnography	6 (4–7)	4–6	37 (40.2%)	NC-I	N
12	In the presence of a biochemical diagnosis of acromegaly and an image of empty *sella turcica*, I assess the ectopic production of GHRH	8 (7–9)	7–9	73 (79.3%)	C-A	Y
13	I request a genetic study in patients with acromegaly and primary hyperparathyroidism	9 (8–9)	7–9	88 (95.7%)	C-A	Y
14	I request a genetic study in patients with acromegaly with macroprolactinoma in a first-degree relative	8 (7–9)	7–9	80 (87%)	C-A	X
15	I request a genetic study in patients with acromegaly younger than 20 years	9 (7–9)	7–9	78 (84.8%)	C-A	X
16	I request a genetic study in a patient with acromegaly and evidence of pheochromocytoma/paraganglioma	9 (8–9)	7–9	83 (90.2%)	C-A	X
17	For the diagnosis of acromegaly in a patient with poorly controlled diabetes, non-increased IGFI and acromegaly suspicion, I determine GH after OGTT	5 (2–7)	4–6	25 (27.2%)	NC-I	N
18	For the diagnosis of acromegaly in a patient with poorly controlled diabetes, non-increased IGFI and acromegaly suspicion, I repeat IGFI after improvement of metabolic control	8 (8–9)	7–9	82 (89.1%)	C-A	Y

*P* percentile, *C* consensus, *NC* non-consensus, *A* agreement, *D* disagreement, *I* indeterminate, *IGFI* insulin-like growth factor I, *ULN* upper limit of normality, *GH* growth hormone, *OGTT* oral glucose tolerance test

*Statement according to guidelines? Y: Yes; N: No; X: Not mentioned in guidelines

**Table 3:** Treatment block

	Statement	Median (P25–P75)	Median range	Participants in median range, n (%)	Consensus	Guidelines*
Primary treatment						
19	I consider that treatment of first choice in acromegaly due to a microadenoma is surgery	9 (9–9)	7–9	89 (96.7%)	C-A	Y
20	I consider that treatment of first choice in acromegaly due to a macroadenoma with compression of the visual pathway is surgery	9 (9–9)	7–9	92 (100%)	C-A	Y
21	I consider that treatment of first choice in acromegaly due to a non-invasive macroadenoma with no visual pathway involvement is surgery	9 (8–9)	7–9	84 (91.3%)	C-A	Y
22♯	I consider that the treatment of choice for acromegaly due to a non-invasive macroadenoma with no visual pathway involvement is the SSA	4 (2–6)	4–6	33 (35.9%)	NC-I	N
22	I consider that the treatment of choice for acromegaly due to a non-invasive macroadenoma with no visual pathway involvement is the SSA instead of surgery	2 (1–3)	1–3	78 (84.8%)	C-D	N
23	I consider that the treatment of choice for acromegaly due to a non-invasive macroadenoma with no visual pathway involvement and with low probability of recovery with surgery is surgical debulking	8 (7–9)	7–9	71 (77.2%)	C-A	Y
24	I consider that treatment of choice in acromegaly due to invasive macroadenoma (Knosp grade III–IV) with no involvement of the visual pathway is SSA	6 (3–8)	4–6	33 (35.9%)	NC-I	Y
Pre-treatment with SSA						
25	I use pre-surgical treatment with SSA when there is delay in surgery	9 (8–9)	7–9	81 (88%)	C-A	Y
26♯	I use pre-surgical treatment with SSA to achieve clinical-biochemical control	6 (4–8)	4–6	25 (27.2%)	NC-I	N
26	I use pre-surgical treatment with SSA to increase the percentage of post-surgical biochemical control	3 (2–5)	1–3	60 (65.2%)	NC-D	N
27	I use pre-surgical treatment with SSA to reduce anesthesia-associated risks	7 (6–9)	7–9	65 (70.7%)	C-A	Y
Second line treatment after surgery failure						
28	In the presence of a persistent disease with potentially resectable post-surgical residual tumor, I prescribe reintervention	8 (6–9)	7–9	69 (75%)	C-A	Y
29	In the presence of a persistent disease with potentially resectable post-surgical residual tumor, I prescribe SSA	6 (5–8)	4–6	29 (31.5%)	NC-I	N
30	In the presence of a persistent disease with unresectable post-surgical residual tumor, I prescribe SSA	9 (9–9)	7–9	92 (100%)	C-A	Y
31	In the presence of a patient with non-cured acromegaly after surgery, with no visible residual tumor by MRI, and IGFI > 1.5 ULN, I initiate treatment with SSA	9 (8–9)	7–9	88 (95.7%)	C-A	N
32	In the presence of a patient with non-cured acromegaly after surgery, with no visible residual tumor by MRI, and IGFI < 1.5 ULN, I initiate treatment with cabergoline	7 (5–8)	7–9	52 (56.5%)	NC-A	Y
33	I use prediction tests for SSA treatment response as a determinant to initiate treatment with these drugs	2 (1–3)	1–3	70 (76.1%)	C-D	N
34	I assess the possible resistance to analogues treatment at 3 months from reaching the maximum dose	8 (6–9)	7–9	69 (75%)	C-A	X
35	I consider there is biochemical resistance to SSA when IGFI decrease is < 50%	7 (4–8)	7–9	49 (53.3%)	NC-A	X
36	I consider there is biochemical resistance to SSA when IGFI decrease is < 25%	8 (7–9)	7–9	73 (79.3%)	C-A	X
37	I consider there is resistance to SSA when an increase of the tumor size occurs	9 (8–9)	7–9	86 (93.5%)	C-A	X
38	In patients with acromegaly with no large residual tumor after surgery, who partially respond to SSA treatment at maximum doses, I preferentially combine SSA with pegvisomant if IGFI is > 2 ULN	8 (7–9)	7–9	82 (89.1%)	C-A	Y
39	In patients with acromegaly with no large residual tumor after surgery, who partially respond to SSA treatment at maximum doses, I preferentially combine SSA with cabergoline if IGFI is < 2 ULN	8 (7–9)	7–9	72 (78.3%)	C-A	Y
40	In the presence of women with a desire for pregnancy and non-cured acromegaly after surgery, with small intrasellar residual tumor, and the rest of the hypophysis function conserved, I initiate medical treatment with SSA	8 (6–9)	7–9	65 (70.7%)	C-A	Y
Radiotherapy						
41	I use radiotherapy in case of aggressive adenoma	9 (8–9)	7–9	83 (90.2%)	C-A	Y
42	I use radiotherapy in patients with unresectable residual tumor	7 (5–8)	7–9	49 (53.3%)	NC-A	N
43	I use radiotherapy in case of unresectable residual tumor, which does not respond to medical treatment	9 (8–9)	7–9	90 (97.8%)	C-A	Y

*P* percentile, *C* consensus, *NC* non-consensus, *A* agreement, *D* disagreement, *I* indeterminate, *IGFI* insulin-like growth factor I, *ULN* upper limit of normality, *GH* growth hormone, *OGTT* oral glucose tolerance test, *SSA* somatostatin analogues

^♯^First round for those items undergoing two rounds

*Statement according to guidelines? Y: Yes; N: No; X: Not mentioned in guidelines

**Table 4:** Follow-up block

	Statement	Median (P25-P75)	Median range	Participants in median range, n (%)	Consensus	Guidelines*
44	I do the first clinical and biochemical control between 1 and 3 months after surgery	9 (8–9)	7–9	87 (94.6%)	C-A	Y
45	In the post-surgical evaluation, I usually request GH after OGTT	7 (5–9)	7–9	55 (59.8%)	NC-A	Y
46	Disease remission exists when the post-surgery random GH value is undetectable (< 1 μg/L)	8 (6–9)	7–9	69 (75%)	C-A	N
47	Disease remission exists when the post-surgery GH value after OGTT is < 1 or ≤ 0.4 μg/L, according to the sensitivity of the GH test	9 (8–9)	7–9	85 (92.4%)	C-A	Y
48	I request the first post-surgical image control between 3 and 6 months after surgery	9 (8–9)	7–9	89 (96.7%)	C-A	Y
49	If 3 months after surgery with apparently complete resection, the IGFI value is > 1.5 and < 2 ULN, I repeat IGFI at 1–3 months without initiating treatment	8 (6–9)	7–9	66 (71.7%)	C-A	Y
50	If 3 months after surgery with apparently complete resection, the IGFI value is > 1.5 and < 2 ULN, I initiate medical treatment	5 (3–8)	4–6	25 (27.2%)	NC-I	N
51♯	If 3 months after surgery with apparently complete resection, IGFI value is > 1.5 and < 2 ULN, I determine GH after OGTT, before initiating treatment	8 (5–9)	7–9	58 (63%)	NC-A	N
51	If 3 months after surgery with apparently complete resection, IGFI value is > 1.5 and < 2 ULN, I determine GH after OGTT, before initiating treatment	8 (5–8)	7–9	67 (72.8%)	C-A	N
52	The initial SSA dose depends upon the initial IGFI concentration; the greater IGFI, the greater initial dose	6 (3–8)	4–6	17 (18.5%)	NC-I	N
53	The initial SSA dose that I use is the maximum dose	3 (2–7)	1–3	49 (53.3%)	NC-D	N
54	After initiating treatment with SSA, the dose is adjusted after the third injection (before the 4th dose)	8 (8–9)	7–9	82 (89.1%)	C-A	X
55	I recommend the patient to have tests done right before the administration of the next SSA dose	9 (8–9)	7–9	81 (88%)	C-A	Y
56	In a patient on SSA treatment and with biochemical control, IGFI determines the possible reduction in dose or administration frequency of the drug	9 (8–9)	7–9	89 (96.7%)	C-A	Y
57	In patients treated with surgery and radiotherapy, from the 5th year onwards, I consider the possibility of decreasing or suspending SSA treatment to evaluate the effect of radiotherapy	8 (7–9)	7–9	77 (83.7%)	C-A	X
58	I request liver function tests after a month from initiating treatment with pegvisomant	9 (8–9)	7–9	87 (94.6%)	C-A	Y
59	I request pituitary MRI after 6 months of pegvisomant treatment	9 (8–9)	7–9	84 (91.3%)	C-A	Y
60	I request pituitary MRI after 6–12 months of SSA treatment	9 (8–9)	7–9	91(98.9%)	C-A	Y
61	In a patient on treatment with 4 mg/week of cabergoline, with no diabetes or hypertension, and with normal initial echocardiogram, I request another echocardiogram after 3 years of treatment	8 (6–9)	7–9	67 (72.8%)	C-A	Y
62	I don´t release an acromegaly patient cured with surgery and with normal hypophysis function	9 (8–9)	7–9	83 (90.2%)	C-A	Y

*P* percentile, *C* consensus, *NC* non-consensus, *A* agreement, *D* disagreement, *I* indeterminate, *SSA* somatostatin analogues, *IGFI* insulin-like growth factor I, *ULN* upper limit of normality, *GH* growth hormone, *OGTT* oral glucose tolerance test

^♯^First round for those items undergoing two rounds

*Statement according to guidelines? Y: Yes; N: No; X: Not mentioned in guidelines

## Discussion

The panel of endocrinologists in our study agreed a variety of statements regarding the diagnosis, treatment and follow-up of acromegaly, which can be used as guidance in the clinical practice (Tables 2, 3, 4). Overall, the assessment of the levels of IGFI is the preferred screening test for acromegaly. If IGFI levels range between 1 and 1.3 of the upper limit of normal (ULN), the test is repeated and GH after oral glucose tolerance test (OGTT) is also assessed. In the clinical practice survey of the first phase of the program, the GH cut off level after a OGTT to discard acromegaly in patients with elevated or equivocal serum IGFI levels at diagnosis was set ≤ 0.4 µg/L by 53% of the surveyed endocrinologists and < 1 µg/L by 37% of them, depending mainly on the sensitivity of the assay. Regarding treatment, surgery is the first choice for patients with microadenoma or macroadenoma with/without optical pathway compression, with pre-surgical SSA being indicated if surgery is delayed and/or to reduce anaesthesia-associated risks. If after an unsuccessful surgery the residual tumor is resectable, reintervention is performed, while SSA are administered if the residual tumor is non-resectable. The first biochemical and clinical controls are performed 1–3 months after surgery, with disease remission being considered if random GH levels are < 1 µg/L or OGTT is < 1 or ≤ 0.4 µg/L (in the clinical practice survey of the first phase of the program, 60% of endocrinologists considered disease remission after surgery when GH after OGTT was ≤ 0.4 µg/L, while 30% considered it when GH after OGTT was < 1 µg/L, depending on the sensitivity of the assay).

Those statements in each block that require further explanation or those for which consensus was not reached, are more widely discussed below.

### Diagnosis

In the case of a patient suspected of having acromegaly, with poorly controlled diabetes and non-increased IGFI, there was no consensus (indeterminate) to determine GH after OGTT for diagnosis of acromegaly (**S17**), since the interpretation of the GH response to the OGTT when diabetes mellitus or impaired glucose tolerance is associated to suspected acromegaly may be difficult [14]; however, panelists agreed to repeat IGFI after improvement of metabolic control in this case (**S18**).

When IGFI values ranged between 1 and 1.3 of the ULN, there was consensus to repeat IGFI (**S3**) and to assess GH after OGTT (**S4**). Guidelines do not advise on how to proceed in this case, however, taking into account that an IGFI value < 1.3 ULN is the limit considered as responsive to medical treatment in clinical trials [15, 16], in view of clinical suspicion, IGFI levels > 1.3 ULN would clearly indicate acromegaly, while a moderate IGFI elevation from 1 to 1.3 ULN should have to be further confirmed.

Faced with a baseline GH = 0.4–1 μg/L and 1.3 ULN < IGFI < 2 ULN, there was consensus to determine GH after OGTT (**S6**). The Spanish guidelines state that a random GH < 0.04 µg/L excludes acromegaly diagnosis [6], but that a random elevated value does not imply its presence. Thus, in this case, although IGFI level is elevated (> 1.3 ULN), the random GH value 0.4–1 μg/L cannot be interpreted, requiring, even more, the determination of GH after OGTT. A GH value < 1 µg/L (or ≤ 0.4 µg/L, depending on the sensitivity of the assay) following documented hyperglycemia during OGTT would rule out acromegaly [5, 6].

Although the panelists agreed to include a colonoscopy within the study of comorbidities (**S8**), following guidelines [5–7], during the regional meetings they argued that invasive tests, such as this one, should not be performed at the time of diagnosis but at a later time during the following year, in order to avoid overwhelming the patients. There was no consensus for including a routine thyroid echography (**S9**) and for the inclusion of a routine polysomnography (**S11**) within the study of comorbidities. As discussed in the meetings, the performance of these tests depends on clinical examination and on the availability of the appropriate equipment, which might be an area to consider for improvement. In fact, the Endocrine Society recommends a thyroid ultrasound only in case of palpable thyroid nodularity [7], and all guidelines recommend, if symptoms are suggestive, to test for sleep apnea with a home overnight oximetry followed by polysomnography [5–7]. In this regard, participants stated during the meeting that they usually use the Epworth sleepiness scale - a short questionnaire to measure daytime sleepiness - [17] and refer the patient to a sleep center when necessary.

There was consensus to assess the ectopic production of GH–releasing hormone (GHRH) in case of biochemical diagnosis of acromegaly and an image of empty *sella* (**S12**). Primary empty *sella* syndrome is frequently associated to GH deficiency; [18] thus, the coexistence of this image with acromegaly might be explained by the existence of GH-secreting pituitary microadenomas or an ectopic pituitary adenoma [19–21], or more likely, by an ectopic secretion of GHRH, usually by a bronchial carcinoid tumor [22–24]. Both of these explanations might apply in the case of a biochemical diagnosis of acromegaly and no tumor image in the pituitary gland MRI [22, 25, 26].

In addition to agreement for requesting a genetic study in patients with primary hyperparathyroidism (**S13**), to rule out multiple endocrine neoplasia type 1 (MEN1) [5, 6], there was also consensus to request a genetic study in patients with macroprolactinoma in a first-degree relative, in patients younger than 20 years, and in patients with evidence of pheochromocytoma/paraganglioma (**S14, 15 and 16**), which are practices not mentioned in guidelines. Acromegaly can be part of familial diseases other than MEN1 [27]. Heterozygous loss-of-function mutations in the aryl hydrocarbon receptor interacting protein (AIP) gene and mutations in the G-protein coupled receptor 101 (GPR101) gene predispose the appearance of young-onset pituitary adenomas [27]. In addition, although the coexistence of acromegaly with paragangliomas or phaeochromocytomas might be coincidental, it could also have common genetic basis, as the recently named “3PAs” syndrome (paragangliomas, phaeochromocytomas and pituitary adenomas) [28, 29].

### Treatment

Consensus was not reached with respect to considering SSA as the treatment of choice in acromegaly due to an invasive macroadenoma (Knosp grade III–IV) with no involvement of the visual pathway (**S24**), since the agreement was on surgical debulking as the treatment of choice for macroadenomas with low probability of recovery with surgery (**S23**). The American Association of Clinical Endocrinologists (AACE) guidelines also state that a role of primary medical therapy with SSA has been suggested in patients with macroadenomas who have no local mass effects and a minimal chance of surgical cure [5]. The Spanish treatment algorithm, when surgery will not cure the disease, gives as alternative treatment choice either SSA or surgery, as well as RT [6].

There was no consensus on the use of pre-surgical treatment with SSA to increase the percentage of post-surgical biochemical control (**S26**), since there are studies showing higher remission rates in patients with pre-surgical SSA than in those with no pre-surgical treatment [30, 31], while other showed no significant differences between groups (at least when a normal level of GH [< 1 µg/L] after OGTT is added to the definition of biochemical remission) [32, 33].

Consensus was not reached for prescribing SSA (**S29**) in case of persistent disease with a potentially resectable post-surgical residual tumor, since the consensus was reached for perfoming reintervention (**S28**), in agreement with Spanish guidelines [6].

Consensus was not achieved to initiate treatment with cabergoline in patients with non-cured acromegaly after surgery, with no visible residual tumor by MRI, and IGFI < 1.5 ULN (**S32**). Cabergoline is specifically recommended in case of modest elevations of IGFI [5–7]; however, in the discussion that took place during the meetings, physicians seemed to prefer using SSA for these cases, and only use cabergoline in combined treatments with the baseline drug.

There was consensus on not to use prediction tests for SSA treatment response as a determinant to initiate treatment (**S33**), given that the utility of acute tests for response prediction is questionable [34, 35].

Biochemical resistance after SSA treatment, which implies non-normal IGFI levels [7], was further defined. Consensus was not reached for an IGFI decrease < 50% (**S35)** but it was for an IGFI decrease < 25% (**S36**), always that IGFI levels stayed elevated, as the definition of SSA biochemical resistance. In addition, there was consensus to consider an increase in tumor size as SSA resistance (**S37**). During the regional meetings the participants commented on the need to add the tumor size to the IGFI level for an overall evaluation of possible SSA treatment resistance, since it seems that only the absence of both responses might be considered as a poor response or resistance [36].

There was consensus to initiate SSA treatment in women with a desire for pregnancy and non-cured acromegalia after surgery, with a small intrasellar residual tumor but preserved function of the rest of the pituitary gland (**S40**), since RT would be contraindicated due to possible hypopituitarism, which would jeopardize fertility [5, 6].

Consensus to use RT was not reached when the statement just mentioned unresectable tumor without further specification (**S42**). Most physicians use medical treatment first, while RT is only used in patients not fully responding to surgical and medical treatments [5–7], being also recommended in cases of uncontrolled macroadenomas [6].

### Follow-up

Consensus was not achieved for requesting GH after OGTT in the post-surgical evaluation (**S45**). Although guidelines consider performing this test [6, 7], only some physicians do it and only when IGFI levels interpretation is not clear (values somewhat high but close to normal limits). The American guidelines recommend measuring a random GH (and an IGFI level) at ≥ 12 weeks from surgery, while GH after OGTT would only be measured in patients with GH > 1 μg/L [7].

The panel considered disease remission when post-surgery random GH is undetectable **(S46)**. Published data and available guidelines consider remission once disease is under control and random GH value is < 1 μg/L. [5, 6, 37] Disease remission is also determined when post-surgery GH value after OGTT is < 1 or ≤ 0.4 μg/L (**S47)**, as stated in guidelines [5, 6, 37].

If 3 months after surgery with apparently complete resection, the IGFI value is > 1.5 and < 2 ULN, there was consensus to repeat IGFI at 1-3 months (**S49**), as according to guidelines [5, 6], and to determine GH after OGTT (**S51**) before initiating treatment; thus, no-consensus on initiating medical treatment directly (**S50**).

There was no consensus with respect to the initial SSA dose; whether it should depend upon the initial IGFI concentration (**S52**) or be the maximum possible dose (**S53**). Guidelines mention conventional doses and approved starting doses [5–7], and when using long half-life SSA, the Spanish guidelines recommend starting at medium dose and adjust according to response [6]. On the other hand, participants agreed to adjust the SSA dose after the third injection (**S54**), which is not mentioned in guidelines.

The study participants agreed to request a pituitary MRI after 6 months of pegvisomant treatment (**S59**), despite the infrequent coincidence of treatment and tumor growth [38], and after 6–12 months of SSA treatment (**S60**), and to request an echocardiogram after 3 years of treatment with cabergoline (4 mg/week) (**S61**).

As agreed by the panel, those patients who are considered cured after surgery (with normal hypophysis function) are not released in the clinical practice (**S62**). Although not explicitly, Spanish guidelines also seem to recommend not to do so, since they state that after confirmation of post-surgery disease control, patients should be followed periodically with serial determinations of GH and IGFI every 6 months at the beginning and yearly later on [6].

In conclusion, overall, most Spanish endocrinologists follow the clinical guidelines; thus, the clinical practice is quite homogeneous and participants agreed on most key aspects to take into consideration when managing acromegaly in the clinical practice. The lack of adherence to some of the guidelines´ recommendations in some practices may be accounted for by certain limitations in resources and another way of managing patients, which made up for such lack, as it occurred in the case of polysomnographies, although other times reflected preferences of Spanish physicians, such as using SSAs instead of cabergoline for modest IGFI elevations.

## References

[1] Melmed S (2009). Acromegaly pathogenesis and treatment. J Clin Invest.

[2] Abreu A, Tovar AP, Castellanos R, Valenzuela A, Giraldo CM, Pinedo AC, Guerrero DP, Barrera CA, Franco HI, Ribeiro-Oliveira A, Vilar L, Jallad RS, Duarte FG, Gadelha M, Boguszewski CL, Abucham J, Naves LA, Musolino NR, de Faria ME, Rossato C, Bronstein MD (2016). Challenges in the diagnosis and management of acromegaly: a focus on comorbidities. Pituitary.

[3] Petrossians P, Daly AF, Natchev E, Maione L, Blijdorp K, Sahnoun-Fathallah M, Auriemma R, Diallo AM, Hulting AL, Ferone D, Hana V, Filipponi S, Sievers C, Nogueira C, Fajardo-Montanana C, Carvalho D, Hana V, Stalla GK, Jaffrain-Rea ML, Delemer B, Colao A, Brue T, Neggers S, Zacharieva S, Chanson P, Beckers A (2017). Acromegaly at diagnosis in 3173 patients from the liege acromegaly survey (LAS) database. Endocr Relat Cancer.

[4] Vilar L, Vilar CF, Lyra R, Lyra R, Naves LA (2017). Acromegaly: clinical features at diagnosis. Pituitary.

[5] Katznelson L, Atkinson JL, Cook DM, Ezzat SZ, Hamrahian AH, Miller KK, American Association of Clinical Endocrinologists (2011). American Association of Clinical Endocrinologists medical guidelines for clinical practice for the diagnosis and treatment of acromegaly–2011 update. Endocr Pract.

[6] Cordido F, Garcia Arnes JA, Marazuela Aspiroz M, Torres Vela E, El Grupo de Trabajo de Neuroendocrinología de la Sociedad Española de Endocrinología y Nutrición (2013). Grupo de Neuroendocrinologia de la Sociedad Espanola de Endocrinologia y Nutricion (Practical guidelines for diagnosis and treatment of acromegaly). Endocrinol Nutr.

[7] Katznelson L, Laws ER, Melmed S, Molitch ME, Murad MH, Utz A, Wass JA, Endocrine S (2014). Acromegaly: an endocrine society clinical practice guideline. J Clin Endocrinol Metab.

[8] Parolin M, Dassie F, Russo L, Mazzocut S, Ferrata M, De Carlo E, Mioni R, Fallo F, Vettor R, Martini C, Maffei P (2017). Guidelines versus real life practice: the case of colonoscopy in acromegaly. Pituitary.

[9] The Delphi method: techniques and applications (2002). https://web.njit.edu/~turoff/pubs/delphibook/delphibook.pdf

[10] Schmitt N (1996). Uses and abuses of coefficient alpha. Psychol Assess.

[11] Cicchetti DV (1994). Guidelines, criteria, and rules of thumb for evaluating normed and standardized assessment instrument in psychology. Psychol Assess.

[12] Dawson B, Trapp RG (2004). Basic & clinical biostatistics (LANGE Basic Science).

[13] Landis JR, Koch GG (1977). The measurement of observer agreement for categorical data. Biometrics.

[14] Kayath MJ, Russo EM, Dib SA, Vieira JG (1992). Do impaired glucose tolerance and diabetes mellitus interfere with the interpretation of the growth hormone response to the oral glucose tolerance test?. Braz J Med Biol Res.

[15] Cannavo S, Ragonese M, Puglisi S, Romeo PD, Torre ML, Alibrandi A, Scaroni C, Occhi G, Ceccato F, Regazzo D, De Menis E, Sartorato P, Arnaldi G, Trementino L, Trimarchi F, Ferrau F (2016). Acromegaly is more severe in patients with AHR or AIP gene variants living in highly polluted areas. J Clin Endocrinol Metab.

[16] Melmed S, Popovic V, Bidlingmaier M, Mercado M, van der Lely AJ, Biermasz N, Bolanowski M, Coculescu M, Schopohl J, Racz K, Glaser B, Goth M, Greenman Y, Trainer P, Mezosi E, Shimon I, Giustina A, Korbonits M, Bronstein MD, Kleinberg D, Teichman S, Gliko-Kabir I, Mamluk R, Haviv A, Strasburger C (2015). Safety and efficacy of oral octreotide in acromegaly: results of a multicenter phase III trial. J Clin Endocrinol Metab.

[17] Johns M (2018) The Epworth sleepiness scale. http://epworthsleepinessscale.com. Accessed 18 Jan 2018

[18] Del Monte P, Foppiani L, Cafferata C, Marugo A, Bernasconi D (2006). Primary “empty sella” in adults: endocrine findings. Endocr J.

[19] Liu W, Zhou H, Neidert MC, Schmid C, Bernays RL, Ni M, Zhou D, Jia W, Jia G (2014). Growth hormone secreting pituitary microadenomas and empty sella—an under-recognized association?. Clin Neurol Neurosurg.

[20] Arzamendi AE, Shahlaie K, Latchaw RE, Lechpammer M, Arzumanyan H (2016). Ectopic acromegaly arising from a pituitary adenoma within the bony intersphenoid septum of a patient with empty sella syndrome. J Neurol Surg Rep.

[21] Hong JF, Ding XH, Wang SS (2012). Coexistence of ectopic pituitary adenoma and empty sella in a patient with acromegaly: a case report and review of literature. Neurol India.

[22] Athanassiadi K, Exarchos D, Tsagarakis S, Bellenis I (2004). Acromegaly caused by ectopic growth hormone-releasing hormone secretion by a carcinoid bronchial tumor: a rare entity. J Thorac Cardiovasc Surg.

[23] Garcia-Luna PP, Leal-Cerro A, Montero C, Scheithauer BW, Campanario A, Dieguez C, Astorga R, Kovacs K (1987). A rare cause of acromegaly: ectopic production of growth hormone-releasing factor by a bronchial carcinoid tumor. Surg Neurol.

[24] Biswal S, Srinivasan B, Dutta P, Ranjan P, Vaiphei K, Singh RS, Thingnam SS (2008). Acromegaly caused by ectopic growth hormone: a rare manifestation of a bronchial carcinoid. Ann Thorac Surg.

[25] Platts JK, Child DF, Meadows P, Harvey JN (1997). Ectopic acromegaly. Postgrad Med J.

[26] Ghazi AA, Amirbaigloo A, Dezfooli AA, Saadat N, Ghazi S, Pourafkari M, Tirgari F, Dhall D, Bannykh S, Melmed S, Cooper O (2013). Ectopic acromegaly due to growth hormone releasing hormone. Endocrine.

[27] Gadelha MR, Kasuki L, Korbonits M (2017). The genetic background of acromegaly. Pituitary.

[28] Xekouki P, Szarek E, Bullova P, Giubellino A, Quezado M, Mastroyannis SA, Mastorakos P, Wassif CA, Raygada M, Rentia N, Dye L, Cougnoux A, Koziol D, Sierra Mde L, Lyssikatos C, Belyavskaya E, Malchoff C, Moline J, Eng C, Maher LJ, Pacak K, Lodish M, Stratakis CA (2015). Pituitary adenoma with paraganglioma/pheochromocytoma (3PAs) and succinate dehydrogenase defects in humans and mice. J Clin Endocrinol Metab.

[29] Denes J, Swords F, Rattenberry E, Stals K, Owens M, Cranston T, Xekouki P, Moran L, Kumar A, Wassif C, Fersht N, Baldeweg SE, Morris D, Lightman S, Agha A, Rees A, Grieve J, Powell M, Boguszewski CL, Dutta P, Thakker RV, Srirangalingam U, Thompson CJ, Druce M, Higham C, Davis J, Eeles R, Stevenson M, O’Sullivan B, Taniere P, Skordilis K, Gabrovska P, Barlier A, Webb SM, Aulinas A, Drake WM, Bevan JS, Preda C, Dalantaeva N, Ribeiro-Oliveira A, Garcia IT, Yordanova G, Iotova V, Evanson J, Grossman AB, Trouillas J, Ellard S, Stratakis CA, Maher ER, Roncaroli F, Korbonits M (2015). Heterogeneous genetic background of the association of pheochromocytoma/paraganglioma and pituitary adenoma: results from a large patient cohort. J Clin Endocrinol Metab.

[30] Abe T, Ludecke DK (2001). Effects of preoperative octreotide treatment on different subtypes of 90 GH-secreting pituitary adenomas and outcome in one surgical centre. Eur J Endocrinol.

[31] Mao ZG, Zhu YH, Tang HL, Wang DY, Zhou J, He DS, Lan H, Luo BN, Wang HJ (2010). Preoperative lanreotide treatment in acromegalic patients with macroadenomas increases short-term postoperative cure rates: a prospective, randomised trial. Eur J Endocrinol.

[32] Losa M, Mortini P, Urbaz L, Ribotto P, Castrignano T, Giovanelli M (2006). Presurgical treatment with somatostatin analogs in patients with acromegaly: effects on the remission and complication rates. J Neurosurg.

[33] Carlsen SM, Lund-Johansen M, Schreiner T, Aanderud S, Johannesen O, Svartberg J, Cooper JG, Hald JK, Fougner SL, Bollerslev J, Preoperative Octreotide Treatment of Acromegaly Study Group (2008). Preoperative octreotide treatment in newly diagnosed acromegalic patients with macroadenomas increases cure short-term postoperative rates: a prospective, randomized trial. J Clin Endocrinol Metab.

[34] de Herder WW, Taal HR, Uitterlinden P, Feelders RA, Janssen JA, van der Lely AJ (2005). Limited predictive value of an acute test with subcutaneous octreotide for long-term IGF-I normalization with Sandostatin LAR in acromegaly. Eur J Endocrinol.

[35] Halperin I, Nicolau J, Casamitjana R, Sesmilo G, Serra-Prat M, Palomera E, Puig-Domingo M (2008). A short acute octreotide test for response prediction of long-term treatment with somatostatin analogues in acromegalic patients. Horm Metab Res.

[36] Colao A, Auriemma RS, Lombardi G, Pivonello R (2011). Resistance to somatostatin analogs in acromegaly. Endocr Rev.

[37] Melmed S, Casanueva FF, Klibanski A, Bronstein MD, Chanson P, Lamberts SW, Strasburger CJ, Wass JA, Giustina A (2013). A consensus on the diagnosis and treatment of acromegaly complications. Pituitary.

[38] Buhk JH, Jung S, Psychogios MN, Goricke S, Hartz S, Schulz-Heise S, Klingebiel R, Forsting M, Bruckmann H, Dorfler A, Jordan M, Buchfelder M, Knauth M (2010). Tumor volume of growth hormone-secreting pituitary adenomas during treatment with pegvisomant: a prospective multicenter study. J Clin Endocrinol Metab.

